# Deficiency of the microglial Hv1 proton channel attenuates neuronal pyroptosis and inhibits inflammatory reaction after spinal cord injury

**DOI:** 10.1186/s12974-020-01942-x

**Published:** 2020-09-05

**Authors:** Xuefei Li, Zhiyuan Yu, Weifeng Zong, Peng Chen, Jia Li, Minghuan Wang, Fengfei Ding, Minjie Xie, Wei Wang, Xiang Luo

**Affiliations:** Department of Neurology, Tongji Hospital, Tongji Medical College, Huazhong University of Science and Technology, Wuhan, 430030 China

**Keywords:** Spinal cord injury, Microglia, Voltage-gated proton channel, Pyroptosis, NLRP3 Inflammasome, Reactive oxygen species, Apoptosis

## Abstract

**Background:**

Spinal cord injury (SCI) causes neurological dysfunction with devastating consequences. SCI pathogenesis is accompanied by inflammasome activation and neuronal damage. But the spatial pattern and the time course of neuronal pyroptosis and apoptosis after SCI should be further elucidated. The microglial voltage-gated proton channel (Hv1) is implicated in reactive oxygen species (ROS)-induced neuronal damage following ischemic stroke. However, there is a lack of quantification on the neuronal pyroptosis and apoptosis associated with microglial Hv1 after SCI.

**Methods:**

We analyzed spatial and temporal characteristics of neuronal pyroptosis and apoptosis following SCI and investigated the effects of Hv1 deficiency on neuronal pyroptosis and the nod-like receptor 3 (NLRP3) inflammasome pathway by using a mouse model of SCI. We tested the effects of Hv1-deficient microglia on ROS production in vivo and examined the relationship between ROS and neuronal pyroptosis in vitro.

**Results:**

We observed that apoptosis was detected closer to the injury core than pyroptosis. The incidence of neuronal apoptosis peaked on day 1 after SCI and occurred before pyroptosis. Hv1 deficiency reduced neuronal apoptosis and NLRP3-inflammasome-mediated pyroptosis, improved axonal regeneration, and reduced motor deficits. SCI led to elevated ROS levels, whereas Hv1 deficiency downregulated microglial ROS generation. In vitro, ROS upregulated neuronal pyroptosis and activated the NLRP3 inflammasome pathway, both of which were reversed by addition of a ROS scavenger. Our results suggested that microglial Hv1 regulated neuronal apoptosis and NLRP3-induced neuronal pyroptosis after SCI by mediating ROS production.

**Conclusion:**

Following SCI, neuronal pyroptosis lasted longer and occurred farther away from the injury core compared with that of neuronal apoptosis. Microglial Hv1 deficiency downregulated microglial ROS generation and reduced apoptosis and NLRP3-induced neuronal pyroptosis. Our findings may provide novel insights into Hv1-associated mechanisms underlying neuronal damage after SCI.

## Introduction

Traumatic spinal cord injury (SCI) results in disability with frequently devastating consequences that affect long-term health and employment status [1]. SCI pathophysiology is initiated by mechanical tissue damage disrupting anatomical and functional integrity of the spinal cord, which contributes to secondary detrimental events. Neuroinflammation plays an important role in secondary neuronal damage following SCI [2]. The inflammatory reaction begins at the lesion site and then expands to surrounding tissue during the secondary phase [3]. A persistent inflammatory microenvironment and the presence of reactive oxygen species (ROS) are potential factors that impede injury repair following SCI [4, 5].

Microglia are major contributors to the inflammatory response after central nervous system (CNS) injury [6]. The voltage-gated proton channel (Hv1) maintains physiological intracellular pH and is abundantly expressed on the surface of microglia [7]. Although Hv1 is specifically expressed in microglia in the nervous system, Hv1 can influence neurons by regulating microglial functions under pathological conductions. Previous studies found that Hv1 was implicated in pathological NADPH-oxidase-mediated ROS generation leading to neuronal apoptosis in ischemic stroke [8, 9]. Our previous study has shown that ROS levels and pro-inflammatory cytokines are reduced in Hv1-deficient (Hv1^−/−^) microglia compared to those in wild-type (WT) microglia after oxygen-glucose deprivation in vitro [10]. However, the contribution of microglial Hv1 in neuronal damage and the underlying molecular mechanisms following SCI remain unknown.

Neuronal death via apoptosis, pyroptosis, or necroptosis is regulated through activities of various host proteins that induce different biological outcomes [11, 12]. In contrast to apoptosis, neuronal pyroptosis is a form of cellular death induced by inflammatory caspases (e.g., caspase-1, caspase-4 and caspase-5, or caspase-11) implicated in ischemic stroke and SCI [13, 14]. However, little is known regarding the spatial and temporal distributions of neuronal pyroptosis and apoptosis following SCI. Cleavage and activation of the pore-forming effector protein, gasdermin D (GSDMD), has been shown to determine pyroptosis initiated by inflammasomes [15]. The nod-like receptor 3 (NLRP3) inflammasome, the well-studied inflammasome, is a multiprotein complex consisting of nod-like receptor 3 (NLRP3), apoptosis-associated speck-like protein (ASC), and an effector pro-caspase-1 [16, 17]. It is unknown whether microglial Hv1 can promote neuronal pyroptosis through the NLRP3 inflammasome after SCI. Here, we used a mouse model to analyze spatial and temporal patterns of neuronal apoptosis and pyroptosis following SCI. We examined whether Hv1 deficiency exerts neuroprotective effects through modulating inflammasome activity and neuronal pyroptosis. Our findings may contribute to the identification and development of a potential therapeutic strategy for individuals with SCI.

## Materials and methods

### Animals

C57BL/6 female mice (wild-type, WT) were obtained from the Laboratory Animal Facilities of Hubei Center (Wuhan, China). Hv1^−/−^ mice were kindly provided by Prof. Long-Jun Wu (Department of Cell Biology and Neuroscience, Rutgers University) and were generated as previous study [18]. The Hv1^−/−^ genotype was confirmed using PCR, Western blotting, and immunofluorescent analysis of tail DNA, spinal cord protein, and tissue sections, respectively. Mice were maintained under a 12-h light/12-h dark cycle at 22 °C and were provided food and water ad libitum. All female mice were 10–12 weeks of age and weighed 20–25 g in weight. WT and Hv1^−/−^ mice were randomly distributed into sham-operation and SCI groups. All experiments and procedures were approved by the Institutional Animal Care and Use Committee of Tongji Medical College, Huazhong University of Science and Technology.

### Spinal cord injury model

Prior to surgery, mice were anesthetized with 2% isoflurane. A T9-11 laminectomy was performed, and the spinal cord was exposed at T10. A 5-g weight was dropped from a height of 11 mm onto the exposed dorsal surface of the spinal cord using a modified NYC impactor (J$K Seiko Electronic, China) [19]. Sham-treated mice underwent a laminectomy at T9-11 without spinal cord injury. The bladder was voided twice daily until the recovery of urinary function post-spinal cord injury (SCI). Locomotor function was evaluated using the Basso Mouse Scale (BMS) [20].

### Tissue preparation

Animals were anesthetized and then perfused transcardially with 0.01 M of phosphate-buffered saline (PBS) at 1, 3, 7, 14, and 28 days after treatment (*n* = 5 for each time point). For Western blotting and ROS detection, spinal cord tissues—including the injury core and approximately 2 cm of surrounding tissue—were rapidly excised and frozen in cooled isopentane. For immunofluorescence, mice were perfused with 4% paraformaldehyde (PFA) in PBS and spinal cord tissues—including the injury core and approximately 2 cm of surrounding tissue—were dissected, fixed in PFA overnight at 4 °C, and then transferred to 30% sucrose in 0.01 M of PBS for 3 days. All the tissue samples were stored at − 80 °C until further use. Ten-micron sections were then prepared for Luxol fast blue (LFB) staining, immunofluorescence, and terminal dexynucleotidyltransferase-mediated dUTP nick-end labeling (TUNEL) assays.

### Luxol fast blue staining

Luxol fast blue (LFB) staining was used to assess lesion volumes after SCI. Briefly, spinal cord tissue sections at 14 and 28 days after treatment were incubated overnight in 0.1% LFB (Servicebio, Wuhan, China) at 60 °C. Sample observations and image acquisitions were performed using a light microscope (BX51, Olympus, Japan).

### Anterograde tracing

Anterograde-tracing was performed to detect axonal regeneration as previously described [21]. Biotinylated dextran amine (BDA; MW,10000; Invitrogen) 10% w/v in sterile PBS was injected into two sites (0.4 μl/ site) of the spinal cord by micropipette. One week later, mice were anesthetized and perfused transcardially with 4% paraformaldehyde. Thirty-micron sections were incubated with Alexafluor 488-conjugated streptavidin (1:500; Invitrogen) for 1 h at room temperature. The images were acquired with a confocal microscope (Olympus, BX51).

### Cell culture

PC12 cells (Cell Storage Center of Wuhan University) were cultured in Dulbecco’s modified Eagle’s medium and Ham’s F-12 (DMEM/F12, Hyclone, USA) supplemented with 10% horse serum (Hyclone, USA), 5% fetal bovine serum (FBS) (Hyclone, USA), and 1% penicillin/streptomycin (Hyclone, USA). Cultures were maintained at 37 °C, 95% air, and 5% CO_2_ in a cell culture incubator (Thermo Fisher Scientific, USA).

### Oxygen-glucose deprivation/reoxygenation and drug treatment

Oxygen-glucose deprivation/reoxygenation (OGD/R) was used to model ischemic injury in vitro, as described previously [22]. Briefly, PC12 cell cultures were washed with ice-cold PBS and culture medium was replaced with glucose-free DMEM (Gibco, USA). Cells were incubated in a hypoxic incubator (Thermo Scientific; USA) at 94% N_2_, 5% CO_2_, and 1% O_2_ for 2 h at 37 °C. Cells were then returned to standard culture medium and incubation conditions (37 °C, 95% air, and 5% CO_2_). The ROS inhibitor, *N*-acetyl cysteine (NAC, Sigma-Aldrich, USA), was dissolved in sterile H_2_O prior to cellular treatments [23].

### Lactate dehydrogenase assay

PC12 cells were cultured in 96-well plates with or without NAC (at concentrations of 0, 10, 20, 30, 50, or 100 μM) for 3, 6, or 24 h. Culture supernatant was collected and analyzed using a commercial lactate dehydrogenase (LDH) kit (Beyotime, China) according to the manufacturer’s instructions.

### Detection of reactive oxygen species

To determine reactive oxygen species (ROS) levels in the spinal cord, tissue samples were homogenized in 10 ml/g of ice-cold PBS as described previously [24]. Homogenates were centrifuged at 10,000×*g* for 10 min at 4 °C. Supernatants were collected and diluted 200-fold in PBS. Then, 100 μl of diluted supernatant was mixed with 100 μl of the molecular probe, 20,70-dichlorodihydrofluorescein diacetate (DCFH-DA, 10 μM; Sigma-Aldrich, USA), and was then transferred to a microplate culture well. To determine PC12 intracellular ROS levels, DCFH-DA (10 μM) was added to cells in serum-free medium. Cells and tissue sample supernatants were incubated with DCFH-DA at 37 °C for 1 h in the dark. ROS levels were measured at 492/520 nm using a multiplate reader (Synergy HT; Biotech, USA).

### Caspase-1 activity

Caspase-1 activity was measured using the Caspase-1 Activity Assay Kit (RD, K111-100) according to the manufacturer’s instructions. Briefly, 2–5 × 10^6^ cells were lysed in 50–100 μl of ice-cold lysis buffer for 10 min and were then centrifuged. Each specimen was incubated in a 96-well plate with Ac-YVAD-pNA (200 μM) at 37 °C for 2 h. The optical density of each specimen was read on a microplate reader (Synergy HT; Biotech, USA).

### Scanning electron microscopy

PC12 cells were fixed in 2.5% glutaraldehyde for 3 h and were then rinsed with PBS. Cells were dehydrated through a graded ethanol series, dried using tertiary butanol, sputter coated with gold, and imaged via scanning electron microscopy.

### Flow cytometry

The PC12 cell apoptotic rate was determined using flow cytometry with an annexin V-FITC/PI apoptosis detection kit (BD Biosciences, 556547) according to the manufacturer’s instructions. Briefly, single-cell suspensions were stained with annexin-V and propidium iodide (PI) at room temperature for 15 min in the dark and were subjected to flow cytometry (BD Biosciences, San Jose, CA). Data were analyzed using FlowJo (TreeStar, Ashland, OR).

### Immunofluorescence

Immunofluorescent staining was performed as previously described [25]. Briefly, tissue sections or cells were blocked using a 10% bovine serum albumin solution with 0.25% triton X-100 for 1 h at room temperature, followed by incubation overnight at 4 °C with primary antibodies. These primary antibodies included rabbit anti-NeuN (1:500, Millipore), rabbit anti-GFAP (1:500, Cell Signaling Technology), rabbit anti-IBA-1 (1:500, Wako Pure Chemical Industries), goat anti-IBA-1 (1:500, Wako Pure Chemical Industries), mouse anti-GSDMD (1:50, Santa-Cruz), mouse anti-ASC (1:200, Santa-Cruz), mouse anti-caspase-1 (1:200, Santa-Cruz), mouse anti-NLRP3 (1:200, AdipoGen), rabbit anti-Hv1 (1:50, Sigma), and mouse anti-8-OHdG (1:200, Abcam). Next, samples were washed in PBS and incubated at room temperature for 1 h with the appropriate secondary antibodies, as follows: FITC-conjugated goat anti-mouse immunoglobulin G (IgG), Cy3-conjugated goat anti-rabbit IgG, Cy3-conjugated rabbit anti goat IgG, Cy3-conjugated rabbit anti-rat IgG, and 488-conjugated donkey anti-goat IgG (Jackson ImmunoResearch, West Grove, PA, USA). Finally, sections were stained with 4,6-diamidino-2-phenylindole (DAPI). Samples were imaged using a confocal microscope (Olympus, BX51).

### TUNEL assay

Apoptosis was detected using the In-Situ Cell Death Detection Kit (TUNEL fluorescence FITC kit, Roche) according to the manufacturer’s instructions. TUNEL staining was performed with fluorescein-dUTP for detection of apoptotic cell nuclei and DAPI for staining of cellular nuclei. The number of TUNEL-positive cells was analyzed via confocal microscopy (Olympus, BX51).

### Western blotting

Tissue samples or PC12 cells were lysed in total protein lysis buffer and protein concentrations were determined using a BCA protein kit (Beyotime, China). Proteins (30 μg) were separated by sodium dodecyl sulfate-polyacrylamide gels (SDS-PAGE) and were transferred to nitrocellulose filters (NCs) or polyvinylidene-difluoride (PVDF) membranes. Membranes were blocked for 1 h at room temperature using 5% non-fat milk in Tris-buffered saline containing 0.1% Tween-20, then incubated overnight at 4 °C with primary antibodies. The following primary antibodies were used: rabbit anti-GSDMD (1:1000, Cell Signaling Technology), mouse anti-Caspase-1 (1:500, AdipoGen), mouse anti-NLRP3 (1:1000, AdipoGen), rabbit anti-ASC (1:1000, Cell Signaling Technology), rabbit anti-IL-18 (1:1000, Abclonal), rabbit anti-Hv1 (1:1000, Sigma), rabbit anti-NOS2 (1:1000; Abclonal), mouse anti-NF-L and anti-NF-H (1:1000; Cell Signaling Technology), rabbit anti-TUJ1 (1:1000, Abcam), mouse anti-MAG (1:1000, Santa Cruz), rat anti-MBP (1:1000, Millipore), rabbit anti-β-actin (1:1000, Servicebio), and rabbit anti-GAPDH (1:1000, Servicebio). Membranes were subsequently incubated with horseradish peroxidase-labeled anti-rabbit, anti-mouse, or anti-rat secondary antibodies (1:5000, Servicebio). Bands were visualized using a Bio-Rad Chemidoc XRS+ imaging system with enhanced chemiluminescent kits (Advansta). Protein levels were determined based on O.D. value using ImageJ software. Protein expression levels were normalized to β-actin or GAPDH internal controls.

### Real-time and semi-quantitative PCR

Spinal cord tissue samples were collected at 1, 3, or 7 days (*n* = 5 for each time point) after surgery and total RNA was isolated using Trizol reagent (Invitrogen). Next, cDNA was prepared using a ReverTra Ace qPCR RT Kit (TOYOBO, Japan). Quantitative real-time PCR (qRT-PCR) was performed using a Real-Time PCR system (BioRad) and SYBR Green PCR Master Mix (TOYOBO, Japan). Then, mRNA expression levels were normalized to those of an endogenous reference gene, glyceraldehyde 3-phosphate dehydrogenase (GAPDH). Semi-quantitative PCR was performed using Quick Taq HS Dye Mix (Japan). PCR products were electrophoresed on a 2% agarose gel and were visualized with GelRed (Biotium; Hayward, CA) and a Gene Genius Bio-Imaging System (Syngene; Cambridge, UK). The following primers were used: interleukin-18 (IL-18) (forward: 5′GGCCGACTTCACTGTACAACCG3′, reverse: 5′GGTCACAGCCAGTCCTCTTACTTC3′), GAPDH (forward: 5′GGTTGTCTCCTGCGACTTCA3′, reverse: TGGTCCAGGGTTTCTTACTCC), Hv1 (forward: 5′-GAGATCCATCTGCCTCCGTTATGAGTG-3′, reverse: 5′-CTGTGTCTCCCTGTGGCTGAG-3′), and β-Geo-R (5′-GACAGTATCGGCCTCAGGAAGATCG-3′).

### Statistical analysis

All data are presented as the mean ± standard error of the mean (SEM). Data were evaluated by one-way or two-way analyses of variance (ANOVAs) with Tukey’s post-hoc tests, when necessary. A value of *p* < 0.05 was considered statistically significant. Statistical analyses were performed using GraphPad Prism 6 (GraphPad Software, Inc., La Jolla, CA).

## Results

### Confirmation of Hv1 gene knockout

**Fig. 1:**
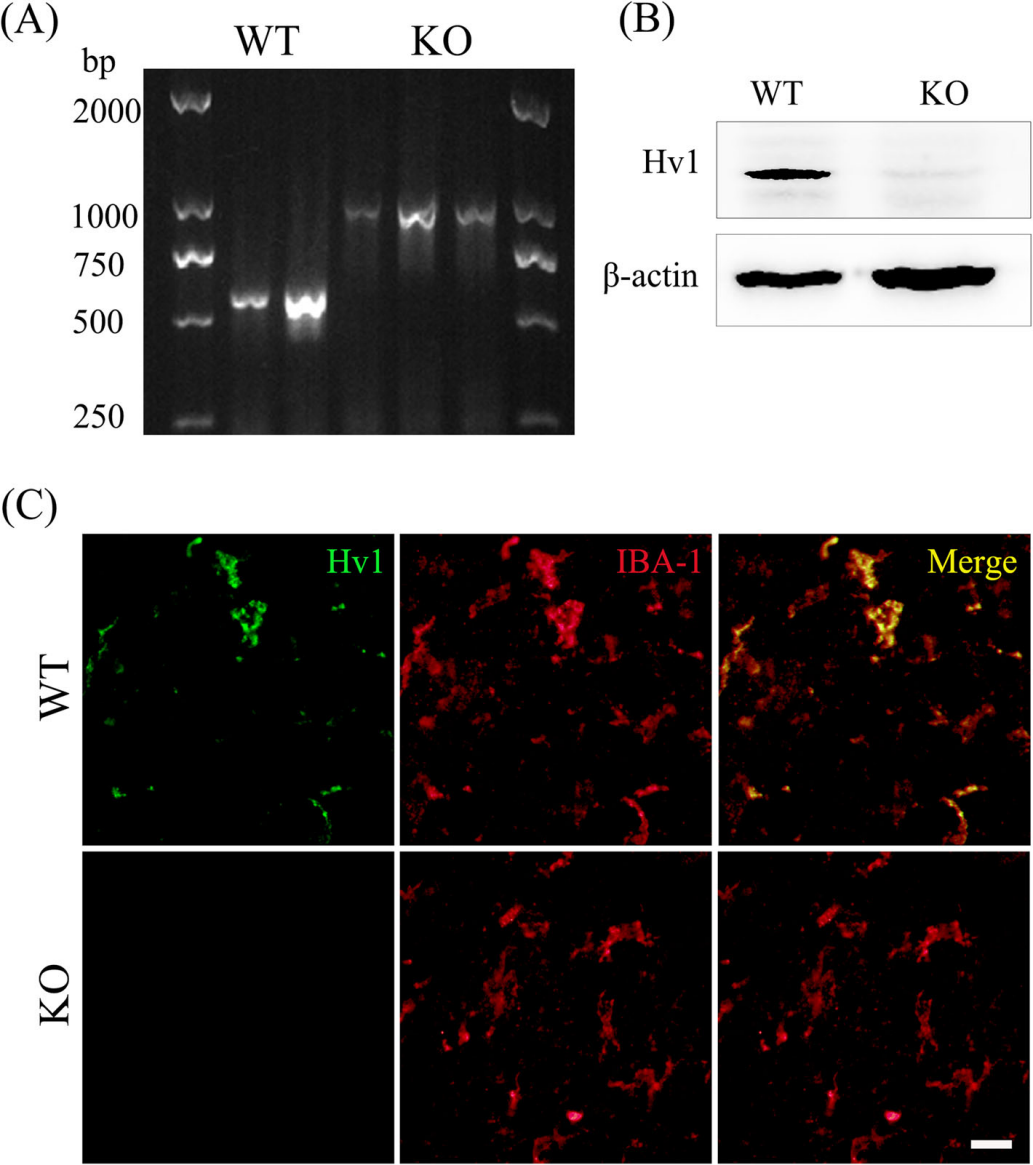
Genotype identification in Hv1^−/−^ mice. **a** Tail DNA analyzed by PCR. **b** Hv1 protein deficiency confirmed by Western blotting. **c** Expression of Hv1 in microglia assessed by double-immunofluorescence labeling with Hv1 (green) and the microglial marker, IBA-1 (red; scale bar, 50 μm). Hv1 was co-localized with the microglial marker, IBA-1, in wild-type (WT) mice. In contrast, Hv1 immunofluorescent signal was rarely observed in Hv1^−/−^ (KO) mice

PCR analysis of genomic DNA revealed a 580-bp band in samples from WT but not Hv1^−/−^ (KO) mice (Fig. 1a). Hv1 protein was not detected in Western-blot analysis of KO mice (Fig. 1b). Immunofluorescent analysis clearly showed Hv1 expression in IBA-1-positive microglia from WT mice, but Hv1 immunoreactivity was rarely detected in microglia from KO mice (Fig. 1c).

### Distinct spatiotemporal patterns of neuronal pyroptosis and apoptosis following SCI; microglial Hv1 deficiency inhibits neuronal pyroptosis and apoptosis

**Fig. 2:**
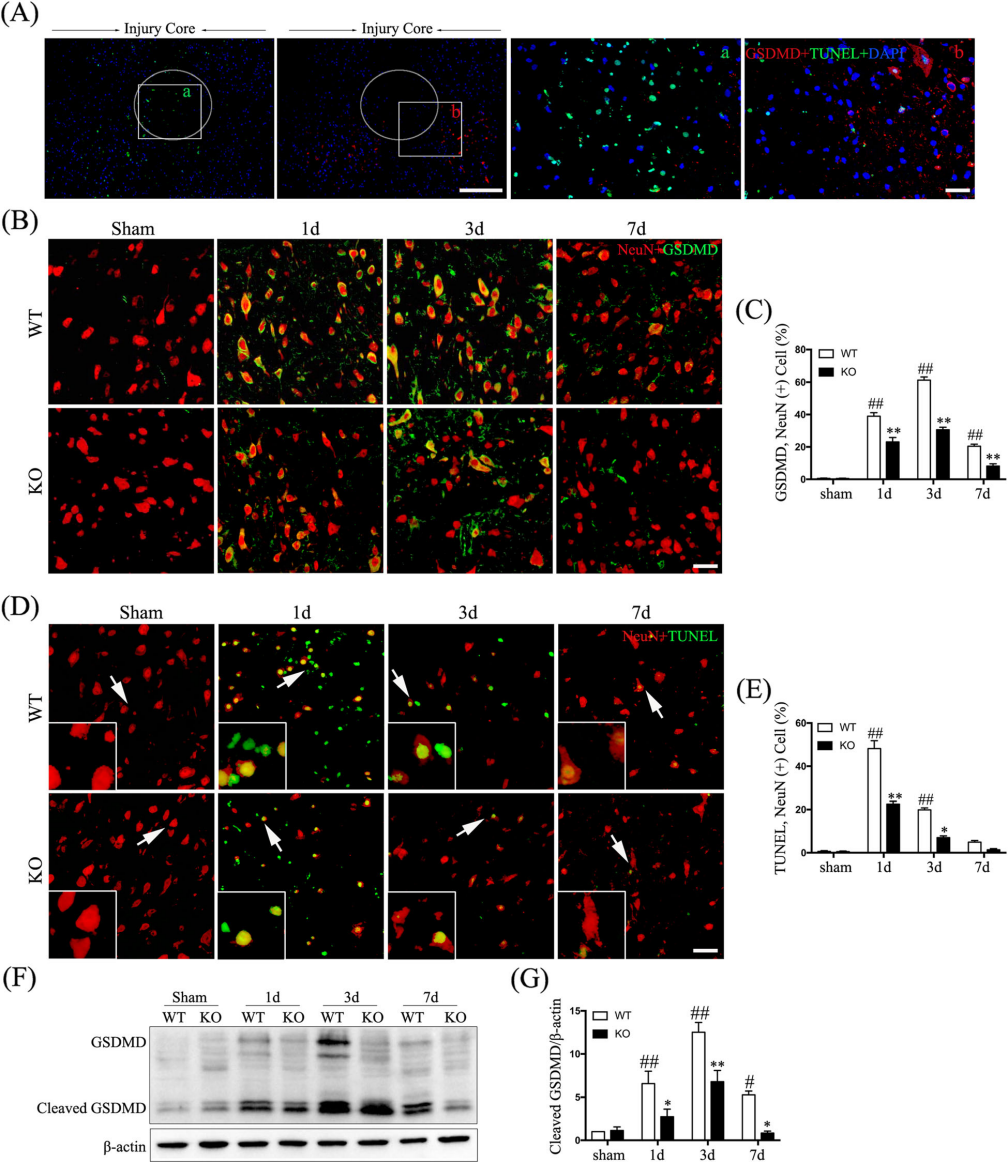
Spatial and temporal distribution of neuronal pyroptosis and apoptosis after SCI and reduced incidence in Hv1 KO mice. **a** Representative images of sections showing the pyroptotic marker GSDMD (red), TUNEL (green), and DAPI (blue; scale bar, 500 μm). **a** The location of the injury core after SCI; b, the area surrounding the injury core after SCI, with apoptosis mainly being located closer to the injury core than pyroptosis (scale bar, 50 μm). **b** Representative confocal images showing GSDMD (green) and NeuN (red) in sham-operated mice and at 1, 3, and 7 days following SCI (scale bar, 50 μm). **c** Quantification of the fraction (%) of GSDMD-positive neurons (GSDMD^+^NeuN^+^ cells/NeuN^+^ cells × 100). **d** Representative images of neurons showing NeuN (red) and TUNEL (green) in sham-operated mice and at 1, 3, and 7 days following SCI (scale bar, 50 μm). **e** Quantification of the fraction (%) of TUNEL-positive neurons (TUNEL^+^ NeuN^+^ cells/ NeuN^+^ cells × 100). **f** Western blotting showing cleaved GSDMD. **g** Quantification of cleaved-GSDMD protein normalized to β-actin from Western blotting (*n* = 5 for each treatment; ^#^*p* < 0.05 ^##^*p* < 0.01, SCI vs. sham treatment; *p < 0.05 **p < 0.01, KO SCI vs. WT SCI)

Increasing evidence indicates acute-phase neuronal pyroptosis and apoptosis participation in the neuropathology of SCI [14]. First, a double-labeling for GSDMD and TUNEL, which are markers of pyroptosis and apoptosis respectively, was performed (Fig. 2a). We found distinct spatial patterns, with TUNEL-positive cells mainly located closer to the injury core than GSDMD-positive cells (Fig. 2a). To better understand the temporal distribution of pyroptotic cells after SCI, we performed a double-labeling of GSDMD and NeuN (Fig. 2b). We found that the frequency of neurons (NeuN^+^ cells) expressing GSDMD was significantly elevated in WT mice after SCI, peaking at 3 days (*p* < 0.01) (Fig. 2c), demonstrating SCI-linked neuronal pyroptosis. To investigate the temporal pattern of neuronal apoptosis, we used a TUNEL assay and observed a significant increase in neuronal apoptosis that peaked at 1-day post-SCI in WT mice (*p* < 0.01) (Fig. 2d, e). Interestingly, the duration of neuronal pyroptosis exceeded that of apoptosis after SCI (Fig. 2c, e).

We further investigated the effects of Hv1 deficiency on neuronal pyroptosis and apoptosis after SCI by comparing the frequencies of neurons (NeuN+) double-labeled by GSDMD or TUNEL in KO vs. WT SCI mice. The numbers of GSDMD-positive (assayed on days 1, 3, and 7 post-treatment; all *p* < 0.01) and TUNEL-positive (assayed on days 1 and 3; *p* < 0.01, *p* < 0.05, respectively) neurons were significantly downregulated in KO SCI mice (Fig. 2c, e). Consistent with these findings, protein levels of cleaved GSDMD, as determined by Western-blot analysis, were significantly decreased in KO mice at 1, 3, and 7 days after SCI (*p* < 0.05; Fig. 2f, g).

### Hv1 deficiency inhibits activation of the NLRP3 inflammasome

**Fig. 3:**
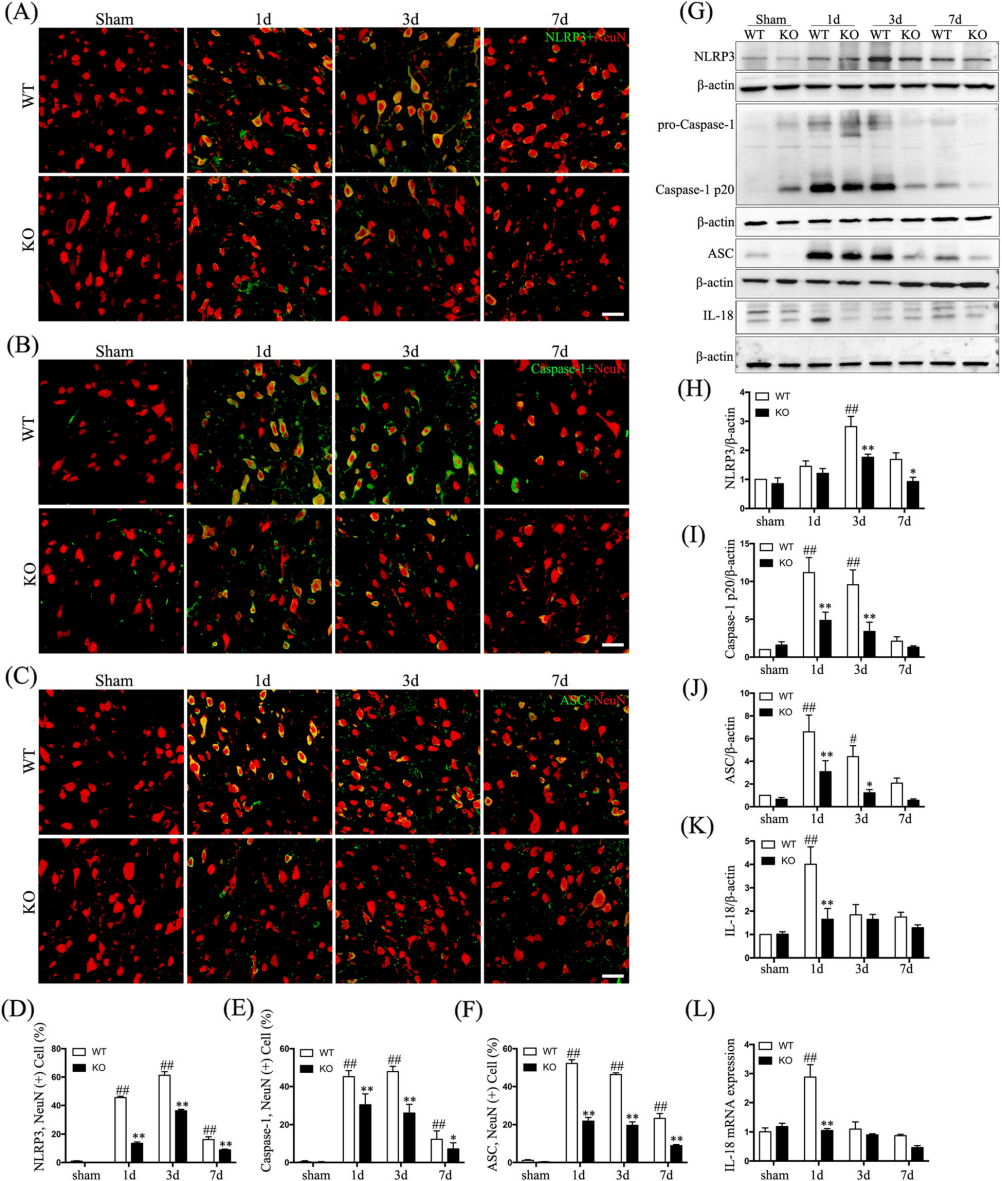
Hv1 deficiency reduces activation of the NLRP3 inflammasome after SCI. **a**–**c** Representative confocal images showing the NLRP3 inflammasome markers (green) NLRP3 (**a**), Caspase-1 (**b**), and ASC (**c**), as well as the neuronal marker, NeuN (red; scale bar, 50 μm). **d**–**f** Quantification of the fraction (%) of **d** NLRP3-positive neurons (NLRP3^+^NeuN^+^ cells/NeuN^+^ cells × 100), (**e**) Caspase-1-positive neurons (Caspase-1^+^NeuN^+^ cells/NeuN^+^ cells × 100) and **f** ASC-positive neurons (ASC^+^ NeuN^+^ cells/NeuN^+^ cells × 100). **g** Western blotting showing NLRP3, caspase-1 p20, ASC, and IL-18 protein levels. **h**–**k** Quantification of Western-blot results for NLRP3 (H), caspase-1 p20 (**i**), ASC (**j**), and IL-18 (**k**) normalized to β-actin. **l** The expression of IL-18 mRNA via real-time PCR. Data are represented as the mean ± SEM (*n* = 5 for each treatment; ^#^*p* < 0.05 ^##^*p* < 0.01, SCI vs. sham treatment; *p < 0.05 **p < 0.01, KO SCI vs. WT SCI)

Upon activation, the NLRP3 inflammasome promotes cleavage of pro-caspase-1 to caspase-1 p20, which then cleaves the pyroptotic factor, GSDMD [26]. We observed differential neuronal upregulation of NLRP3, caspase-1, and ASC proximal to the injury site after SCI in WT compared to that in sham-operated mice (Fig. 3a–c). Hv1 KO mice had lower numbers of NLRP3-positive neurons (at 1, 3, and 7 days; all *p* < 0.01), caspase-1-positive neurons (at 1, 3, and 7 days; *p* < 0.05), and ASC-positive neurons (at 1, 3, and 7 days; *p* < 0.01) (Fig. 3d–f). Furthermore, we analyzed NLRP3, ASC, and caspase-1 p20 protein levels via Western blotting (Fig. 3a). At 1 and 3 days following SCI, caspase-1 p20 and ASC levels were significantly downregulated in KO mice compared to those in WT mice (*p* < 0.05), and NLRP3 levels in KO mice were strikingly lower at both 3 and 7 days following SCI (*p* < 0.05) (Fig. 3h–j). Importantly, NLRP3 inflammasome activation has also been reported to promote maturation and release of IL-18 [27]. As shown in Fig. 3g and k, expression of IL-18 protein was upregulated in WT animals at 1 day after SCI compared to that of sham-operated mice and was comparatively decreased in KO mice (all *p* < 0.01). Next, qRT-PCR analysis confirmed that IL-18 upregulation was significantly increased in WT vs. KO mice at 1 day after SCI (*p* < 0.01) (Fig. 3l). Hv1 deficiency therefore significantly attenuated activation of the NLRP3 inflammasome, which is consistent with the effect of Hv1 deficiency on neuronal pyroptosis.

### Hv1 deficiency attenuates ROS production

**Fig. 4:**
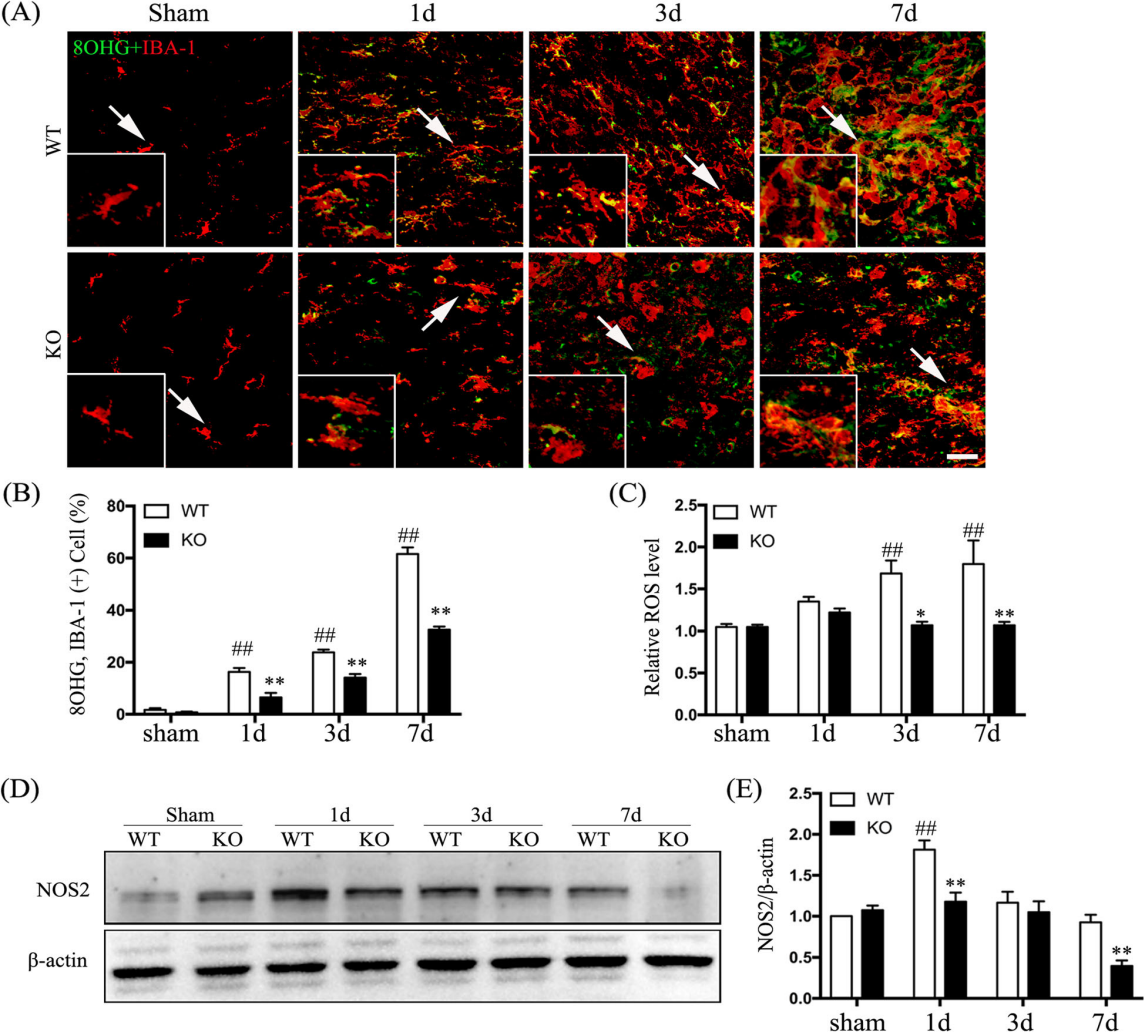
Hv1 deficiency suppresses microglial ROS generation after SCI. **a** Representative confocal images of coronal sections showing IBA-1 (red) and 8 hydroxyguanosine (8-OHG) (green) at different time points post-SCI (scale bar, 50 μm). **b** Quantification of the fraction (%) of 8-OHG and IBA-1 double-positive cells (8-OHG^+^ IBA-1^+^ cells/IBA-1^+^ cells × 100) in sham-operated mice and at 1, 3, and 7 days following SCI. **c** Quantitative analysis of relative ROS levels (DCFH-DA fluorescent intensity normalized to that in sham mice). **d** Western blotting showing NOS2 and β-actin. **e** Quantification of NOS2 normalized to β-actin (from Western blotting). Data are represented as the mean ± SEM (*n* = 5 for each treatment; ^##^*p* < 0.01, SCI vs. sham treatment; *p < 0.05 **p < 0.01, KO SCI vs. WT SCI)

In mouse models, secondary damage following SCI is closely linked to excessive microglial ROS generation [28]. We used an 8-OHG-specific antibody to detect oxidized nucleic acids caused by cellular ROS [29]. 8-OHG immunoreactivity was mainly localized in microglia (IBA-1^+^ cells) and the percentage of 8-OHG-positive microglia was significantly elevated in the boundary zone of the injury core after SCI compared to that in sham-operated mice (Fig. 4a). Furthermore, the fraction of 8-OHG-positive microglia after SCI was markedly lower in KO mice compared to that in WT mice (*p* < 0.01) (Fig. 4b). We further observed increased ROS levels in WT mice at both 3 and 7 days after SCI compared to those in sham-operated mice (*p* < 0.01), while SCI elicited lower ROS generation in Hv1^−/−^ mice compared to that in WT mice (*p* < 0.05) (Fig. 4c). Finally, Western-blot analysis showed that NOS2 protein levels were significantly lower in KO mice compared to those in WT mice at 1 and 7 days after SCI (*p* < 0.01) (Fig. 4d, e). These findings suggest an important role of microglial Hv1 in ROS generation after SCI.

### ROS regulates pyroptosis in PC12 cells

**Fig. 5:**
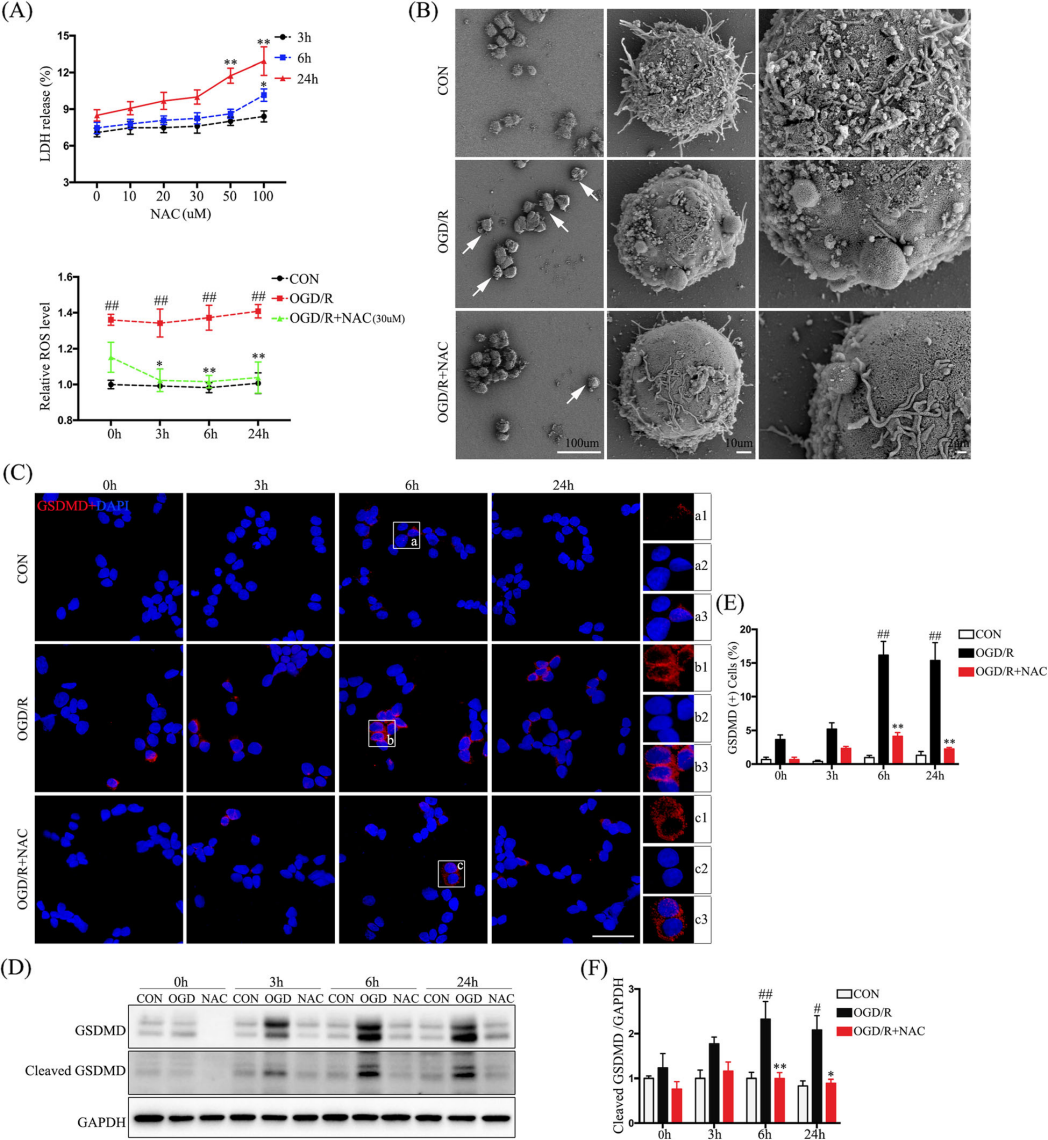
ROS regulates pyroptosis in PC12 cells. **a** Quantification of LDH release indicating PC12 cell damage and relative ROS levels (DCFH-DA fluorescent intensity normalized to that in control cells). **b** Representative scanning electron microscopy image showing morphological changes accompanying PC12 cell pyroptosis induced by OGD/R. **c** Representative confocal images of PC12 cells showing the pyroptotic marker, GSDMD (red), as well as DAPI (blue). **d** Western-blot analysis of GSDMD. **e** Quantification of the percentage of GSDMD-positive cells (GSDMD^+^DAPI^+^ cells/DAPI^+^ cells × 100). **f** Quantification of cleaved GSDMD (from Western blotting) normalized to GAPDH. Data are represented as the mean ± SEM (*n* = 5 for each treatment; ^##^*p* < 0.01, OGD/R vs. control; *p < 0.05 **p < 0.01, OGD/R + NAC vs. OGD/R)

To directly address whether Hv1 regulation of ROS generation after SCI is relevant to neuronal pyroptosis and apoptosis, we next employed a PC12-cell OGD/R oxidative stress model in vitro. We first measured cellular oxidative stress using DCFH-DA and observed significantly elevated ROS levels after OGD/R (*p* < 0.01) (Fig. 5a). To clarify the relationship between ROS and neuronal pyroptosis, we used the ROS scavenger, NAC. We first performed LDH assays to measure NAC-induced cytotoxicity in PC12 cells. As shown in Fig. 5a, NAC elicited dose- and time-dependent release of LDH. We also found that 30 μM of NAC significantly reduced ROS levels in the OGD/R model (*p* < 0.05). Therefore, we used a 30 μM concentration of NAC to suppress ROS generation in subsequent experiments.

Pyroptosis is characterized by cellular swelling and the emergence of large bubbles from the plasma membrane [30]. Consistent with these published observations, using scanning electron microscopy, we observed these morphological characteristics in PC12 cells undergoing OGD/R (Fig. 5b). To determine whether PC12 cell pyroptosis was regulated by ROS, we compared GSDMD expression by immunofluorescence (IF) and Western-blot analyses among untreated control, OGD/R, and OGD/R + NAC-treated cells (Fig. 5c, d). Cleaved GSDMD protein was markedly upregulated in PC12 cells at 6 and 24 h after OGD/R and was inhibited by NAC (*p* < 0.05) (Fig. 5e, f). ROS therefore contributed to PC12 cell pyroptosis in our OGD/R model in vitro.

### ROS promotes PC12 cell apoptosis

**Fig. 6:**
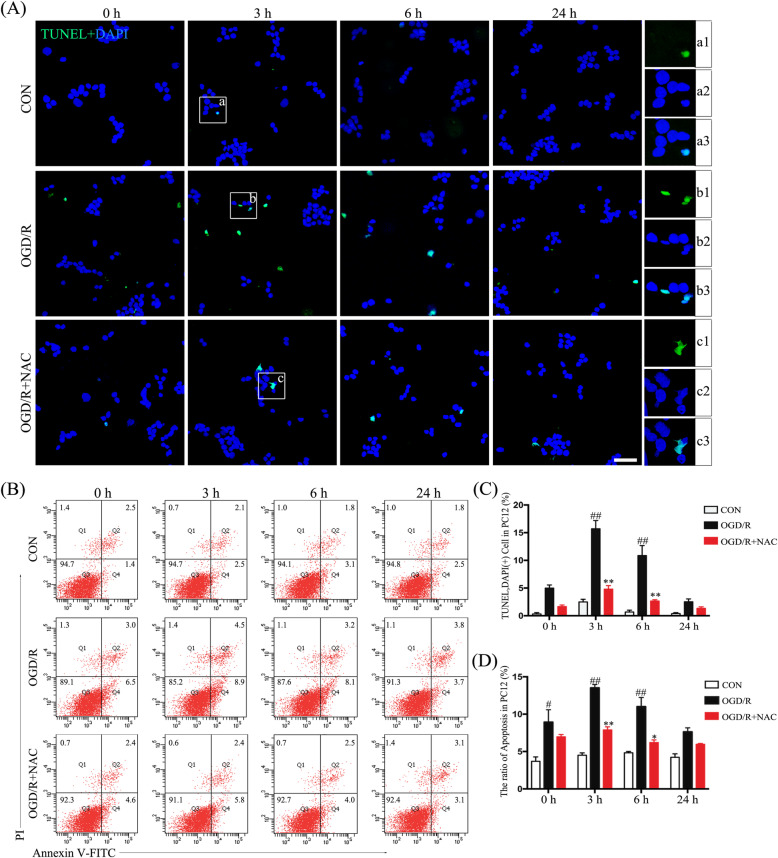
ROS modulates apoptosis of PC12 cells. **a** Representative images of PC12 cells showing TUNEL (green) and DAPI (blue; scale bar, 50 μm). **b** FACS analysis of PC12 cell apoptosis using Annexin V/PI kit. **c** Quantification of the percentage of TUNEL-positive cells (TUNEL^+^DAPI^+^ cells/DAPI^+^ cells× 100). **d** Ratios of apoptotic PC12 cells among different treatment groups (total ratio of apoptosis = Q2 + Q4). Data are represented as the mean ± SEM (*n* = 5 for each treatment; ^#^*p* < 0.05 ^##^*p* < 0.01, OGD/R vs. control; *p < 0.05 **p < 0.01, OGD/R + NAC vs. OGD/R)

To further investigate temporal distinctions in neuronal pyroptosis and apoptosis, as observed following SCI, we measured PC12 cell apoptosis using flow cytometry with annexin V/PI and TUNEL staining (Fig. 6a, b). Apoptosis was significantly increased at 3 and 6 h after OGD/R (*p* < 0.05) (Fig. 6c, d), which was earlier than observations for pyroptosis. We also found that PC12 cell apoptosis was reduced in OGD/R-treated cells exposed to NAC (*p* < 0.05) (Fig. 6c, d).

### ROS scavenger rescues activation of the NLRP3 inflammasome in PC12 cells after OGD/R

**Fig. 7:**
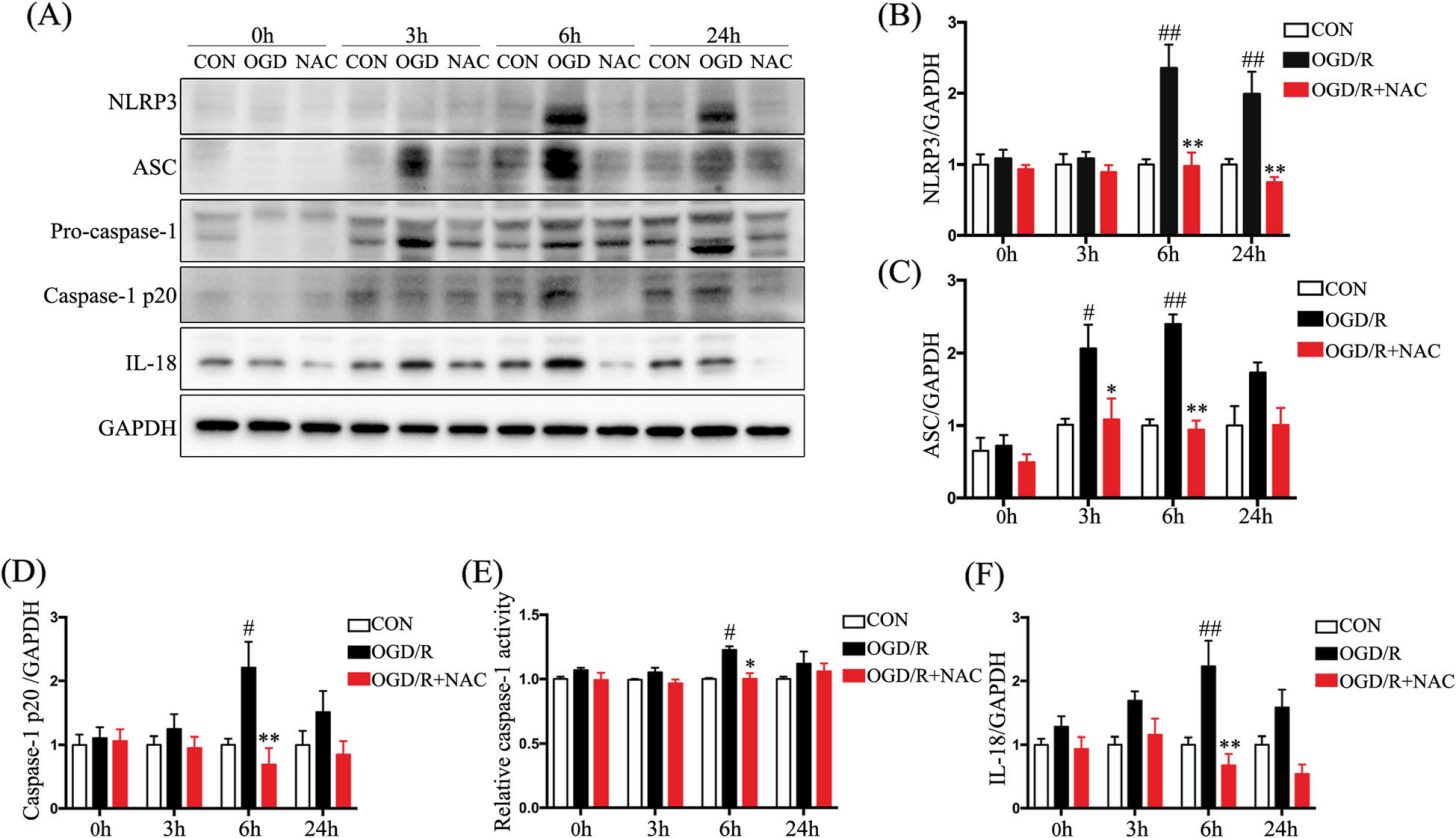
A ROS scavenger rescues ROS-mediated NLRP3 inflammasome activation. **a** Western blotting showing NLRP3, ASC, caspase-1 p20, and IL-18 levels. **b**–**d** Quantification of Western-blot analysis for NLRP3 (**b**), ASC (**c**), and caspase-1 p20 (**d**) normalized to GAPDH. **e** Relative caspase-1 activity after different treatments. **f** Quantification of Western-blot analysis for IL-18 normalized to GAPDH. Data are represented as the mean ± SEM (*n* = 5 for each treatment; ^#^*p* < 0.05 ^##^*p* < 0.01, OGD/R vs. control; *p < 0.05 **p < 0.01, OGD/R + NAC vs. OGD/R)

We used the OGD/R PC12 cell model to investigate the relationship between ROS and the NLRP3 inflammasome pathway. Activation of the NLRP3 inflammasome (consisting of NLRP3, ASC, and pro-caspase1) was determined by Western blotting (Fig. 7a). Levels of NLRP3, ASC, and caspase-1 p20 were significantly upregulated after OGD/R (*p* < 0.05) (Fig. 7b–d), while levels of NLRP3, ASC, and cleaved caspase-1 were significantly downregulated by the addition of NAC (*p* < 0.05) (Fig. 7b–d). Caspase-1 activity was directly measured and shown to be enhanced at 6 h after OGD/R but was inhibited by addition of NAC (*p* < 0.05) (Fig. 7e). We also found that upregulation of IL-18 protein level at 6 h after OGD/R was suppressed by NAC (*p* < 0.01) (Fig. 7a, f). These results suggest that ROS is essential for activation of the NLRP3 inflammasome pathway in PC12 cells in the OGD/R model.

### Hv1 deficiency promotes axonal regeneration and the recovery of motor functions after SCI

**Fig. 8:**
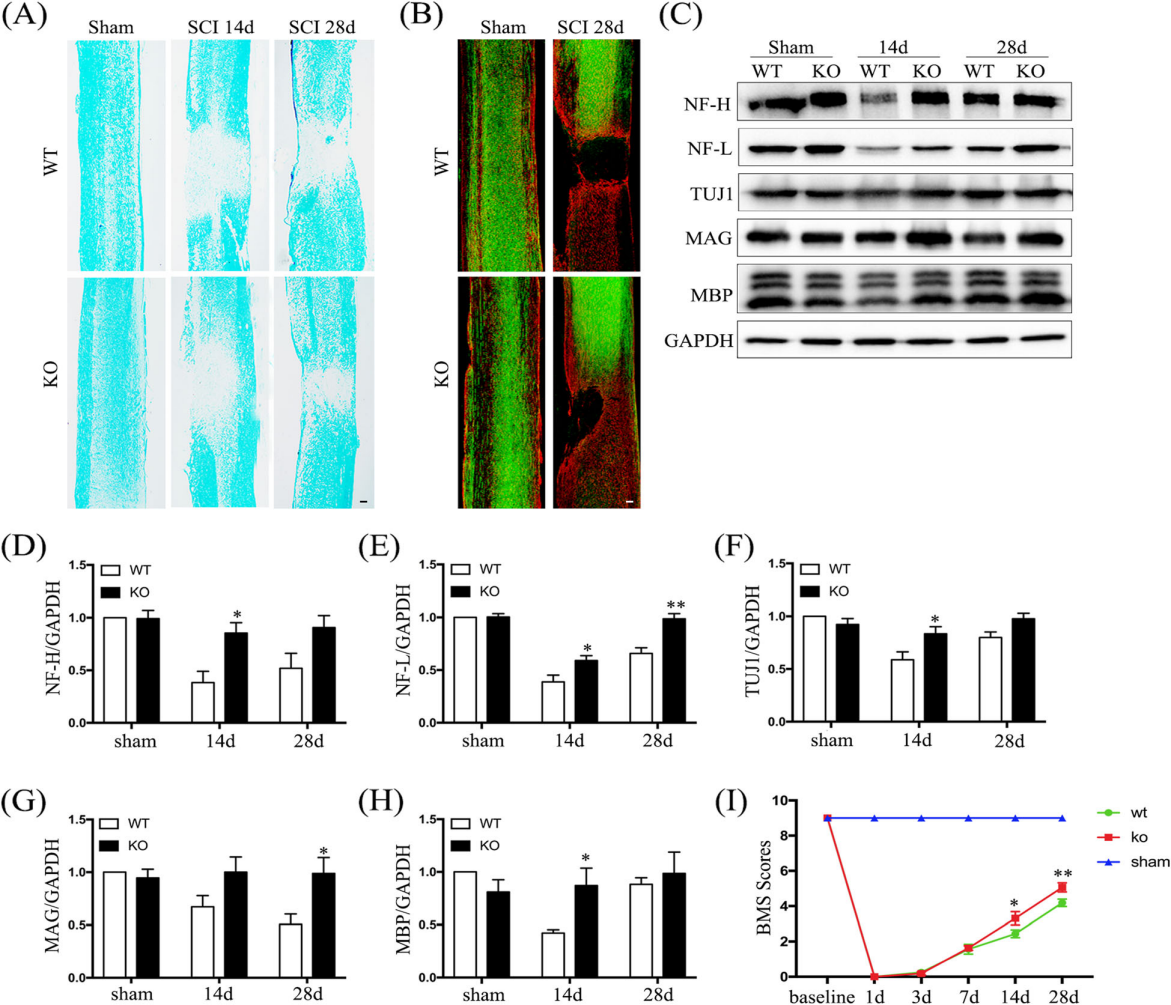
Hv1 deficiency promotes axonal regeneration and recovery of motor function. **a** Representative image of Luxol fast-blue staining on days 14 and 28 after SCI (scale bar, 200 μm). **b** Representative image of GFAP staining (red) and BDA-labeled axons (green) in sham-operated mice and at 28 days after SCI in WT and KO mice (scale bar, 200 μm). **c** Western blotting showing NF-H, NF-L, TUJ1, MAG, and MBP levels in sham-operated mice and at 14 and 28 days after SCI. **d**–**h** Quantification of Western-blot results for NF-H (**d**), NF-L (**e**), TUJ1 (**f**), MAG (**g**), and MBP (**h**) normalized to GAPDH. Values are represented as the mean ± SEM (*n* = 5 for each treatment). **i** Analysis of Basso Mouse Scale (BMS) scores before and after SCI in WT and KO mice (*n* = 8 for each genotype; *p < 0.05 **p < 0.01, KO SCI vs. WT SCI)

To further investigate the effects of Hv1 deficiency on lesion volumes and axonal recovery following SCI, we performed LFB staining, GFAP staining, and anterograde tracing (Fig. 8a, b). After 14 and 28 days after SCI, LFB staining demonstrated that lesion volumes were smaller in Hv1^−/−^ mice than that in WT mice (Fig. 8a). As shown in Fig. 8b, many integrated BDA-labeled axons were observed in sham-operated groups. After 28 days after SCI, few BDA-labeled axons were visualized in the caudal side of the injury in WT groups; however, in Hv1^−/−^ mice, some axonal sprouts passed through astrocyte scar borders and extended into the spared grey matter. Then, we further assessed levels of the axonal markers, neurofilament-L (NF-L) and neurofilament-H (NF-H), and the neuronal-specific marker, βIII-tubulin (TUJ1), via Western blotting (Fig. 8c). In Hv1^−/−^ mice, NF-H and TUJ1 were increased at day 14, while NF-L was increased at 14 and 28 days after SCI (*p* < 0.05) (Fig. 8d–f). Myelin-associated glycoprotein (MAG) is key to maintaining axon-glial integrity and myelin basic protein (MBP) is a general myelin marker. MAG and MBP levels were significantly increased at 28 and 14 days following SCI in KO mice, respectively, compared to those in WT mice (*p* < 0.05) (Fig. 8g, h). Motor function recovery was evaluated using the BMS scoring system [20]. BMS scores were markedly better for KO mice compared to those for WT mice at 14 and 28 days after SCI (*p* < 0.05) (Fig. 8i). These observations are consistent with Hv1 deficiency promoting axonal regeneration and recovery of motor function after SCI.

## Discussion

The present study found distinct spatial and temporal patterns of neuronal pyroptosis and apoptosis after SCI and showed that Hv1 deletion significantly attenuated NLRP3-inflammasome-mediated neuronal pyroptosis and apoptosis as well as facilitated myelin-axon regeneration paralleled by improved motor function. These findings suggest that the effects on ROS-mediated modulation of NLRP3 inflammasome activation underlie protection against neuronal damage from Hv1 deficiency. To our knowledge, the present study is the first to elucidate spatial and temporal differences in neuronal pyroptosis and apoptosis during early-stage SCI. Moreover, this work is the first to reveal the underlying mechanism by which Hv1 deficiency attenuates neuronal pyroptosis after SCI.

Apoptosis has long been recognized as the predominant in a wide variety of neurological conditions [31]. Recently, findings found that another special death mechanism different from apoptosis participated in the CNS pathology, which has been named as pyroptosis in 2001 [32, 33]. Pyroptosis is characterized by cellular swelling [30], formation of 10–14-nm GSDMD pores [34], caspase-1-dependent cell death, and release of inflammatory cytokines such as IL-18 [33]. More recently, neuronal pyroptosis—a gasdermin-mediated programmed necrotic cell death—has been reported to be linked to traumatic brain injury [33], cerebral ischemia/reperfusion injury [25], and SCI [14]. In the present study, we showed that neuronal expression levels of the pyroptosis marker GSDMD and the inflammatory factor IL-18 were significantly upregulated in vivo SCI model in mice and in vitro OGD/R model in neurons mimicking SCI, which are consistent with the previous opinion that neuronal pyroptosis contributed to neuroinflammation after SCI [33]. Accumulating studies showed that apoptosis and pyroptosis could both occur in neuron during the pathology of CNS diseases [25, 33]. However, few researches further focused on the spatiotemporal patterns of neuronal pyroptosis and apoptosis. Here, for the first time, we reported that the peak of neuronal apoptosis prior to that of neuronal pyroptosis and the duration of pyroptosis was much longer than that of apoptosis in vitro and in vivo experiments. These results suggest that in contrast to apoptosis, timely intervention to inhibit neuronal pyroptosis following SCI is feasible. Thus, identifying a means of regulating neuronal pyroptosis after SCI is potentially valuable.

Hv1 channel has been reported to regulate neuronal death in the pathogenesis of ischemic stroke [8]. Our present study also showed that neuronal apoptosis following SCI was reduced in Hv1^−/−^ mice relative to that in WT mice. However, information on microglial Hv1-mediated neuronal pyroptosis remains unclear. Interestingly, our study is the first to discover that the expression of pyroptosis in neurons was lower in Hv1^−/−^ mice compared with that in WT mice after SCI. Microglia and macrophages, the first cells to be recruited in response to injury, are the most important modulators of secondary damage after SCI [35] and activated microglia contribute to ROS generation [36]. Hv1 channel, functionally expressed in microglia, has been reported to induce NADPH oxidase (NOX)-mediated ROS generation [8]. In the current study, we observed upregulation of microglial ROS generation after SCI, which was significantly attenuated in Hv1^−/−^ mice in the acute period after SCI. This finding is consistent with our previous report that microglial Hv1 deficiency could attenuate ROS level [10]. Emerging evidence showed that ROS signaling was the key in the initiation of pyroptotic death involving diverse diseases [37, 38]. It was proposed that pyroptosis in hemorrhagic shock and resuscitation was downregulated through reducing ROS production by mitochondria [37]. In another study, ischemia-reprerfusion-induced pyroptosis remarkably relied on ROS which was significantly inhibited by ROS scavenger NAC [38]. In line with these previous researches, our in vitro data further showed that ROS-induced pyroptosis by OGD/R model in neurons was obviously rescued by NAC. Therefore, we reasonably believe that microglial Hv1 channel exacerbated neuronal pyroptosis through enhancing ROS production after SCI.

Unlike apoptosis, pyroptosis is an inflammatory cell death requiring the activation of caspase-1 [39]. Caspase-1 activation is modulated by protein complexes termed inflammasomes. Inflammasomes can be divided into different types, such as NLRP1, NLRP2, NLRP3, NLRC4, NLRP6, NLRP7, and AIM2 [14]. Among these inflammasomes, NLRP3 inflammasome expression is markedly upregulated following SCI, thus playing an important role in spinal cord tissue after SCI [40]. Furthermore, Zendedel et al. [41] found that NLRP3 was expressed in neurons, microglia, and astrocytes, and that neurons represented the major source of NLRP3. Here, we showed that the expressions of NLRP3, ASC, and caspase-1 p20 in neurons were significantly increased after SCI in mice, the findings of which are consistent with those of a previous study [14]. In addition, NLRP3, ASC, and caspase-1 levels were lower in Hv1^−/−^ mice, suggesting that microglial Hv1 is a potential target for modulating the NLRP3 inflammasome. NLRP3 is triggered in response to diverse stimuli including microparticles, pore-forming toxins, ATP, and ROS [42]. ROS acts as a second messenger that plays a significant role in NLRP3 inflammasome activation [43]. The activation of NLRP3 recruits ASC and promotes the activation of caspase-1, thus processing cytokines to their active forms to ultimately induce pyroptotic death [27]. We detected OGD/R activation of the NLRP3 inflammasome pathway in neurons in vitro, which again was inhibited by NAC, thus revealing a key role of ROS in this process. These results suggest that microglial Hv1-induced ROS generation regulates NLRP3-inflammasome-mediated pyroptosis after SCI.

Neuronal death in the injured area following SCI may contribute to disruption of neural circuitry, ultimately leading to functional impairment [44]. Hence, inhibition of neuronal delayed death is critical to promoting axonal regeneration after SCI [45]. Additionally, reducing neuroinflammation may create a favorable micro-environment for neuronal and myelin repair [46]. Collectively, our findings suggest that inhibition of Hv1 can promote myelin/axonal regeneration and concomitantly improve motor function after SCI. Based on our current results, we propose that microglial Hv1 deficiency may serve as a promising strategy to improve SCI outcomes.

## Conclusions

In summary, our study provides novel insight into the spatiotemporal patterns of neuronal apoptosis and pyroptosis after SCI, and reveals a potential efficacy of targeted inhibition of microglial Hv1 to prevent neuronal pyroptosis. Accordingly, the development of therapeutic interventions designed to inhibit microglial Hv1 after SCI may be beneficial in afflicted patients.

## Data Availability

Information about the experimental methods, animal model, and data supporting the conclusions of current study are included within the article.

## Abbreviations

SCI: Spinal cord injury
ROS: Reactive oxygen species
WT: Wild-type
KO: Knockout
GSDMD: Gasdermin D
NLRP3: Nod-like receptor 3
ASC: Apoptosis-associated speck-like protein
BMS: Basso mouse scale
PBS: Phosphate-buffered saline
PFA: Paraformaldehyde
LFB: Luxol fast blue
BDA: Biotinylated dextran amine
TUNEL: Terminal dexynucleotidyltransferase-mediated dUTP nick end labeling
DMEM/F12: Dulbecco’s modified eagle’s medium and Ham’s F-12
FBS: Fetal bovine serum
OGD/R: Oxygen-glucose deprivation/reoxygenation
NAC: *N*-acetyl cysteine
LDH: Lactate dehydrogenase
DCFH-DA: Dichlorodihydrofluorescein diacetate
SEM: Standard error of the mean
PI: Propidium iodide
IF: Immunofluorescence
DAPI: 4,6-Diamidino-2-phenylindole
SDS-PAGE: Sodium dodecyl sulfate-polyacrylamide gel
NC: Nitrocellulose filter
PVDF: Polyvinylidenedifluoride
qRT-PCR: Quantitative real-time PCR
IL-18: Interleukin-18
ANOVA: Analysis of variance
NF-L: Neurofilament-L
NF-H: Neurofilament-H
TUJ1: βIII-tubulin
MAG: Myelin-associated glycoprotein
MBP: Myelin basic protein
